# Case Report: The Clinical Toxicity of Dimethylamine Borane

**DOI:** 10.1289/ehp.8287

**Published:** 2005-08-12

**Authors:** Yu-Tse Tsan, Kai-Yu Peng, Dong-Zong Hung, Wei-Hsiung Hu, Dar-Yu Yang

**Affiliations:** Department of Emergency, Taichung Veterans General Hospital, Taichung, Taiwan, Republic of China

**Keywords:** chemical decontamination, dimethylamine borane, neurotoxicity, polyneuropathy, semiconductor plating

## Abstract

Context: Dimethylamine borane (DMAB) is a reducing agent used in nonelectric plating of semiconductors. Exposures are usually through occupational contact. We report here four cases of people who suffered from work-related exposure to DMAB.

Case presentation: Three patients exposed to DMAB decontaminated immediately by drinking a lot of water; they reported dizziness, nausea, diarrhea 6–8 hr later. The other patient did not decontaminate at once, and he suffered from more severe symptoms, including dizziness, nausea, limb numbness, slurred speech, irritable mood, and ataxia 13 hr later. Magnetic resonance imaging showed symmetric lesions with hyperintensity on T2WI and FLAIR in bilateral cerebellar dantate nuclei. This patient was readmitted to the hospital due to difficulty in walking and climbing 18 days after exposure. Lower leg weakness and drop foot were found bilaterally. A nerve conduction study revealed polyneuropathy with motor-predominant axonal degeneration. This patient receives regular outpatient followups and still walks with a clumsy gait and has difficulty with hand-grasping activity.

Discussion: This case study demonstrates that DMAB is highly toxic to humans through any route of exposure, and dermal absorption is the major route of neurotoxicity. DMAB induces acute cortical and cerebellar injuries and delayed peripheral neuropathy.

Relevance: Further investigation of the toxic mechanism of DMAB is warranted. Early decontamination with copious water is the best current treatment for exposure to DMAB.

Dimethylamine borane [DMAB, dimethylamine-borane complex; (CH_3_)_2_NHBH_3_, CAS no. 74-94-2] is a strong reducing agent and is an important chemical in the semiconductor industry (Jagannathan and Krishnan 1993). DMAB is a white, crystalline solid with a molecular weight of 58.92 g/mol and melting point of 33–36°C. The chemical structure of DMAB is shown in Figure 1 (BASF 2004).

DMAB is toxic and hazardous to the environment (BASF 2004). It is an irritant and is corrosive to the skin and mucosa (BASF 2004). However, to our knowledge, there have been no published reports, to date, of human exposure. Here we report a case of occupational DMAB exposure that caused significant neurotoxicity. We also found three other cases of occupational DMAB exposure during our field investigation.

## Case Presentation

**Figure 2:**
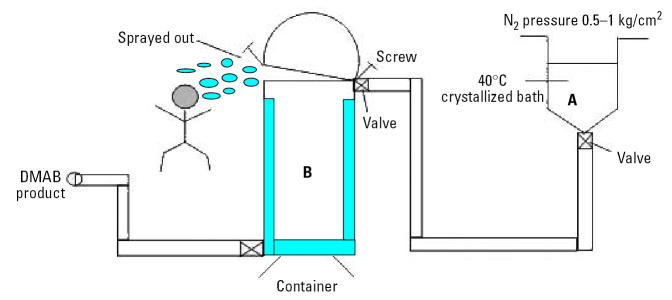
Diagram showing how the patient was exposed to DMAB during the production process. *A*, Tank where DMAB is produced. *B*, container holding DMAB product; one of the screws on the lid of the container came loose, and liquid DMAB sprayed out over the face, head, and trunk of the worker.

A 36-year-old, healthy male was accidentally sprayed over the face and trunk with the liquid form of DMAB (Figure 2). He kept on working and did not take a shower until > 1 hr later. He developed dizziness, nausea, vomiting, sore throat, limb numbness, slurred speech, slow motion, lack of concentration, and ataxia by the next morning, 13 hr after exposure. He was admitted to a local hospital, where a normal brain computerized tomogram was noted. Because of worsening clinical conditions, including “masked” face, irritability, awkwardness, and rocking from side to side while sitting on the bed, he was transferred to our hospital 3 days later. Physical examination revealed some abnormal neurologic findings. The patient was oriented as to time and place but was easily distracted. His speech was slurred. Normal muscle power was noted for all four limbs. He could stand on a wide base with assistance but deviated to both sides when attempting a tandem gait. Impairment on finger-to-nose and heel-to-knee tests was also noted. He denied any medical problems such as hypertension, diabetes, and neurologic diseases. He smoked one pack of cigarettes per day and drank alcohol occasionally.

A routine laboratory work-up including complete blood cell count, electrolytes, blood sugar, and hepatic and renal function tests was performed. Mild hyperventilation, with arterial blood gas of pH 7.510, partial pressure of carbon dioxide 30.6 mm Hg, partial pressure of oxygen 100 mm Hg, and bicarbonate 24.7 mmol/L, was found. No drug history, including use of herbal medicine, was noted for the last 3 months. Urinalysis did not detect any illegal drugs, central nervous system-acting drugs, or other medications. Normal blood and urine lead, mercury, and aluminum levels were also noted.

**Figure 3:**
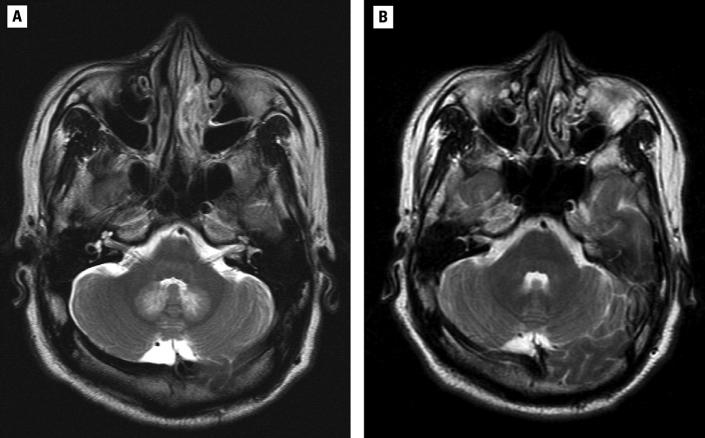
MRIs of the patient. (*A*) Symmetric increase in signal intensity at bilateral cerebellar periventricular area on T2WI (9 February 2004). (*B*) Previous cerebellar dantate nuclei region hyperintensity on T2WI has subsided (17 March 2004).

Eight days after chemical exposure, the patient’s electroencephalogram (EEG) revealed diffuse background slowing, indicative of a mild diffuse cerebral dysfunction. Tests of nerve conduction velocity (NCV) for the left-side limbs were normal. Brain magnetic resonance imaging (MRI) on the eighth day showed a symmetric increase in signal intensity on FLAIR (fluid-attenuated inversion recovery), T2WI (T2-weighted intensity), and DWI (diffusion-weighted images), but low signal intensity on T1WI without postcontrast enhancement at bilateral cerebellar periventricular areas (Figure 3A). A steroid was prescribed for treatment of the possible acute inflammatory effects on neurons. The patient was discharged with stable neurologic function after 6 days of observation.

The pateint was readmitted to our neurology outpatient clinic 18 days after chemical exposure due to difficulty in walking and climbing. Physical examination revealed that the deep tendon reflexes of both knees were areflexic. Muscle power was mildly decreased in the distal and proximal parts of the upper right leg. Lower leg weakness and drop foot were also found bilaterally with muscle power of grade 2/5 in the right foot and 3/5 in the left foot. A nerve conduction study on the 29th day after poisoning showed decreased NCV and complex muscular action potential (CMAP) amplitudes for the left median, left ulnar, left peroneal, and left tibial nerves. H-reflex was absent bilaterally. Sensory conduction and sensory evoked potential tests of the nerves of the upper left and lower left limbs were normal. A brain MRI on the 37th day after poisoning showed that the previous lesions in the cerebellar dentate nuclei region had subsided (Figure 3B).

With active physical therapy, the patient could walk straight on a wide base 2 months after poisoning. No dysmetria was noted on the finger-to-nose test, but heel or toe gait was impaired. The muscle power was grade 3/5 in the flexor and extensor of the right foot; 4/5 in the flexor and extensor of the left foot, and others were all 5/5. Weakness in the flexor and extensor of both feet still remained. A repeat EEG was normal. A repeat NCV study revealed no change in polyneuropathy with motor predominant axonal degeneration. The patient receives regular outpatient followups. He still walks with a clumsy gait and has difficulty with hand-grasping activity.

## Field Investigation

We performed a field investigation to study the character and mechanism of chemical exposure. According to the statement of the facility manager, the factory produces only DMAB. The liquid sprayed on the patient was 97% DMAB. The other 3% was decomposed materials including boric acid, borates, hydrogen, and dimethylamine (DMA). DMAB was the only toxic substance at the workplace.

There were three other workers with a history of DMAB contamination. Their data are summarized in Table 1 (cases 2–4). They all suffered from minor intoxication without any residual neurologic sequelae.

## Discussion

To our knowledge, the human toxicity of DMAB has never been reported in the literature. In the BASF material safety data sheet, DMAB is noted to be toxic and hazardous to the environment (BASF 2004). It is harmful if swallowed or absorbed through the skin. Both vapor and solid can cause eye, skin, and respiratory tract irritation. Studies of animals exposed to high doses of DMAB have demonstrated injury to the kidneys, liver, adrenals, lungs, and central nervous system (BASF 2004). Our patients reveal that DMAB is highly toxic to humans through any route of exposure. The major route of toxicity is dermal absorption. Gastrointestinal symptoms occur the first 6–12 hr after exposure, but the toxicity of DMAB seems to be limited if prompt decontamination is performed immediately after exposure. Delayed decontamination after DMAB exposure in our patient did lead to severe toxicity, including acute cerebral and cerebellar dysfunction and delayed polyneuropathy. The cerebral and cerebellar toxicity of DMAB was temporary, as evidenced in the patient’s serial MRI and EEG examinations and clinical manifestations. The mechanism of central nervous system lesions is unknown, but from the study of serial MRI, transient demyelination, axonal degeneration, or neuron damage might be suggested (Bradley 1986). According to the clinical neurologic manifestations and EEG upon admission, we also suggest that some cortical dysfunction may have been induced by DMAB, though it was a negative finding on the image study 8 days after exposure.

Delayed peripheral neuropathy was the second important presentation in this case of DMAB poisoning. The decreased muscle power of the four limbs developed progressively during the 3 weeks after DMAB exposure. We verified the polyneuropathy with axonal degeneration by serial EEG/NCV studies.

DMAB easily decomposes to boric acid, borates, hydrogen, and DMA (BASF 2004). DMA is also toxic by inhalation, ingestion, and intravenous routes. Gases or vapors from aqueous solutions may cause irritation, conjunctivitis, and corneal damage. Inhalation may cause coughing, nausea, and pulmonary edema [American Conference of Government Industrial Hygienists (ACGIH) 1991], but no systemic effects of DMA intoxication from industrial exposure have been reported (Ballantyne et al. 1985). Boric acid is well absorbed through the gastrointestinal tract, open wounds, and serous cavities. It causes gastrointestinal symptoms (nausea, vomiting, and diarrhea) and dermal effects (erythema, desquamation). The central nervous system effects are less common in intoxication by boric acid in adults. Boric acid causes headache, lethargy, restlessness, weakness, and seizure, but cerebellar lesions have not been reported (Kiesche-Nesselrodt and Hooser 1990; Locatelli et al. 1987; Mack 1984; Siegel and Wason 1986; Von Burg 1992). Hydrogen is usually nontoxic when inhaled, but it can displace oxygen, leading to oxygen deficiency in a confined space. In a rat study, repeated administration of DMAB produced rather severe central nervous system lesions (BASF 2004). The liquid or vapor form of DMAB, in concentrations of ≥97%, might be a reason for central and peripheral neurotoxicity.

## Conclusion

DMAB intoxication can lead to acute cortical and cerebellar lesions and delayed polyneuropathy. Early and prompt decontamination is indicated in an occupational setting. Further research is needed regarding the mechanism of DMAB poisoning.

## Figures and Tables

**Figure 1:**
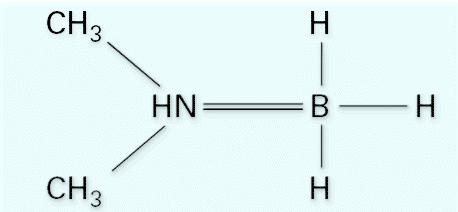
Chemical structure of DMAB [(CH_3_)_2_NHBH_3_] (BASF 2004).

**Table 1:** Data of four male workers exposed to DMAB.

Case no.	Age (years)	Route of entry	Decontamination	Symptom onset time	Symptoms	Subside/sequelae
1	36	Sprayed on the face and head	Not immediately (1 hr later)	12 hr	Altered consciousness, irritable, had difficulty walking and climbing, dizziness, slurred speech, limb numbness, nausea, vomiting, gastrointestinal upset	Symptoms persisted
2	32	Sprayed over the whole body	Took a shower immediately	6 hr	Dizziness, nausea, vomiting, and had diarrhea 3 times	Symptoms subsided the next morning
3	28	Ate a particle of DMAB with rice meal	Drank a lot of water immediately	6 hr	Dizziness, nausea, vomiting, and had diarrhea 5 times	Recovered 1 day later
4	40	Sprayed on face and mouth	Took a shower immediately	8 hr	Dizziness, nausea, vomiting, and had diarrhea once	Recovered 1 day later
